# Changing attitudes towards annual influenza vaccination amongst staff in a Tertiary Care Irish University Hospital

**DOI:** 10.1007/s11845-021-02636-w

**Published:** 2021-05-13

**Authors:** Emma C. Kearns, Ian Callanan, Ann O’Reilly, Aisling Purcell, Niamh Tuohy, Siobhan Bulfin, Angela Smyth, Emer Bairead, Susan Fitzgerald, Eoin Feeney, Sarmad Waqas

**Affiliations:** 1 Department of Surgery, St. Vincent’s University Hospital, Dublin, Ireland; 2 Department of Clinical Audit, St. Vincent’s University Hospital, Dublin, Ireland; 3 Department of Occupational Health, St. Vincent’s University Hospital, Dublin, Ireland; 4 Department of Quality and Patient Safety, St. Vincent’s University Hospital, Dublin, Ireland; 5 Department of Microbiology, St. Vincent’s University Hospital, Dublin, Ireland; 6 Department of Infectious Diseases, St. Vincent’s University Hospital, Dublin, Ireland

**Keywords:** Attitude of health personnel, Clinical audit, Infection control, Occupational health, Patient safety

## Abstract

**Background:**

Healthcare workers are encouraged annually to get vaccinated against influenza. This year in view of COVID-19 pandemic, attitudes of HCWs towards vaccination are particularly important. A cross-sectional study was completed to understand how to best encourage and facilitate the vaccination of HCWs based on the previous years’ findings.

**Methods:**

An online survey was disseminated to all hospital staff via electronic channels. The clinical audit sphinx software was used for data collection and analysis.

**Results:**

The total number of responses was *n* = 728, almost double the rate from 2018 (*N* = 393). A total of 78% (*N* = 551) of participants were vaccinated last year. A total of 94% (*N* = 677) of participants reported their intention to be vaccinated this year. The main barriers listed were being unable to find time (32%, *N* = 36), side effects (30%, *N* = 33) and thinking that it does not work (21%, *N* = 23). The most popular suggestions for how to increase uptake were more mobile immunisation clinics (72%, *N* = 517) and more information on the vaccine (50%, *N* = 360). A total of 82% of participants (*N* = 590) agreed that healthcare workers should be vaccinated, with 56% (*N* = 405) agreeing that it should be mandatory. Of the participants who were not vaccinated last year (*N* = 159), 40% (*N* = 63) agreed that COVID-19 had changed their opinion on influenza immunisation with a further 11% (*N* = 18) strongly agreeing.

**Discussion:**

In light of the increasing number of survey participants, more staff were interested in flu vaccination this year than ever before. The COVID-19 pandemic has had some influence on staff’s likelihood to be vaccinated. Feasibility of immunisation and education posed the largest barriers to HCW vaccination.

## Background

Healthcare workers (HCWs) are encouraged annually to get vaccinated against influenza to protect themselves as well as patients. Despite this, uptake rates remain usually low, e.g., at less than 40% in Europe [1]. Influenza presents a significant burden to healthcare, with seasonal epidemics and pandemics [2]. The World Health Organisation (WHO) estimates that it accounts for 250,000 to 500,000 of deaths per year globally [3].

Healthcare-associated influenza infection presents a public health threat. HCWs are at increased risk of exposure to respiratory pathogens such as influenza [2]. In a Canadian study, over a 6-year period, 17.3% of cases of influenza were healthcare-associated [4]. In a Japanese study, 20–30% of cases of influenza were accounted for by healthcare workers as evidenced by syndromic surveillance [5]. Furthermore, HCWs do not only acquire influenza, but they can also transmit and spread the infection to patients [2]. Staff can be asymptomatic, but remain infectious with a positive nasopharyngeal swab [6].

Vaccines remain the most effective tool for preventing the flu [2]. The influenza vaccine has evolved over 60 years, ensuring effective protection while also maintaining a good safety and tolerability profile [7]. WHO continuously surveys which influenza strains are isolated from the previous season to determine the composition of the quadrivalent vaccine each year.

In Ireland, the uptake rates of flu vaccination have been increasing in recent years with 58.32% in 2019/2020 compared with 53.2% in 2018/2019 in hospital staff [8]. While this increase is encouraging, these figures still fall well below targets. In 2020/2021, the Irish National Public Health organisation, the Health service executive (HSE), aims to achieve a target of 75% flu vaccine uptake among healthcare workers [9].

This year in view of COVID-19 pandemic, attitudes of HCWs towards vaccination are particularly important. Whilst a vaccine against COVID-19 is still in development, it is crucial that barriers to vaccination are identified and addressed early, in preparation for a COVID-19 vaccine.

The aims of this study were to gain insight into how to best encourage and facilitate staff to be vaccinated, with the ultimate goal of maximizing uptake rates in 2020/2021.

## Methods

A mixed methods survey was created with a variety of question types (quantitative, qualitative, Likert-based questions). A copy of this survey is included in Appendix 1. Similar surveys have been undertaken in our institute in 2015, 2016 and 2018. Two additional questions were included this year pertaining to the COVID-19 pandemic. Previously, the survey was disseminated to staff in paper form. This year, in light of infection control precautions, it was entirely online and disseminated to staff (*n* = ~ 3500) via email and mobile communications. As an incentive to complete the survey, participants were entered in a raffle to win vouchers. Staff were given 48 h to complete the survey. The software “Clinical Audit Sphinx” was used for data collection and analysis. Data is available on request from the authors. Results of this year’s survey were compared to the 2018 results. In comparing results, each year describes an influenza winter season. For example, the 2018 results describe answers for the 2018/2019 winter season.

The survey was commenced after approval from the Clinical Audit Committee (Approval Number 28510). This department oversees all quality improvement projects in our hospital and a separate ethics committee approval is not required in our institute if a quality improvement project is approved by the Clinical Audit Committee. This project focused on healthcare staff and did not involve patients or members of the public.

## Results

In total, 728 hospital staff completed the survey, a response rate of approximately 20%. This is an increase in response rate of 9.6%, with *n* = 393 responses in 2018.

### Demographics

The majority of participants were female (79%, *n* = 569) and in the age categories of 30–39 years (30%, *n* = 217) and 40–49 years (30%, *n* = 213). Focusing on the breakdown of professions, the responses were spread across the divisions between doctors (20%, *n* = 146), nurses (20%, *n* = 144), allied health professionals (23%, *n* = 168), management/administration (24%, *n* = 169), general support services (5%, *n* = 37) and other patient care, i.e. healthcare assistants or HCAs (7%, *n* = 51).

### Staff being vaccinated

Seventy-eight percent (*n* = 551) of the survey participants in 2020 received the influenza vaccine in 2019, compared to 80% (*n* = 316) surveyed in 2019 who had got the vaccine in 2018. Reasons for getting the vaccine included: it being recommended (69%), protecting ourselves (79%), protecting patients (59%), protect colleagues (56%), protecting family members (64%) and because other staff members were doing it (8%). Ninety-four percent of participants reported that they intend to get the vaccine this year, compared to 95.9% in 2018.

### Staff not being vaccinated

Of the staff who did not get vaccinated last year (*n* = 159) as per the survey responses, staff in administration were the least likely to be vaccinated (30%, *n* = 48), while nurses (19%, *n* = 30) and AHPs (18%, *n* = 29) were second, followed by other patient care (13%, *n* = 20), doctors (11%, *n* = 18) and general support services (8%, *n* = 13). Of those who do not intend to get vaccinated this year (*n* = 45), management/administrative staff also made up the majority of participants (33%, *n* = 15), followed by nurses (24%, *n* = 11), other patient care (16%, *n* = 7), general support services (13%, *n* = 6), doctors (7%, *n* = 3) and AHPs (4%, *n* = 2).

### Barriers to vaccination

**Fig. 1:**
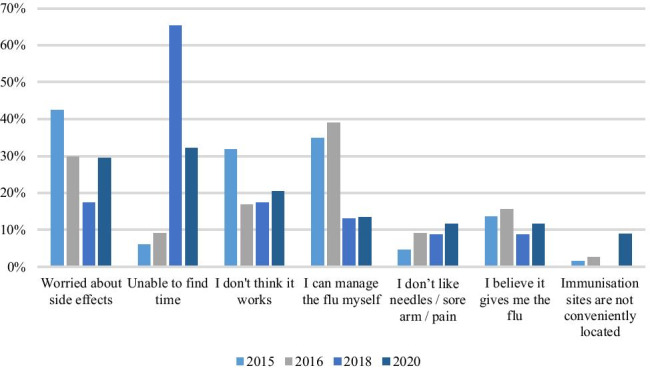
Barriers to being vaccinated

Reasons for not being vaccinated included: being unable to find time (32%, *n* = 36), side effects (30%, *n* = 33), not thinking that it works (21%, *n* = 23), believing they can manage the flu themselves (13%, *n* = 15), not liking needles/sore arm/pain (12%, *n* = 13), believing that it gives them the flu (11%, *n* = 12) and that immunisation sites are not conveniently located (9%, *n* = 10). This presented a stark difference to previous years’ results with 65% (*n* = 15) of staff reporting side effects as their reason in 2018, while 17% (*n* = 4) reported being unable to find time (Fig. 1). In 2015 and 2016, the most common barriers were being worried about side effects at 42% and 30%, respectively, and being able to manage the flu themselves at 35% and 39%, respectively. Being unable to find the time was much less important to staff in 2015 and 2016, at 6% and 9%, respectively.

### Likert-based questions

We included four Likert-based questions this year. Two had been asked in previous years, with a further two questions added pertaining to COVID-19.

### I believe healthcare workers should be immunised against influenza

**Fig. 2:**
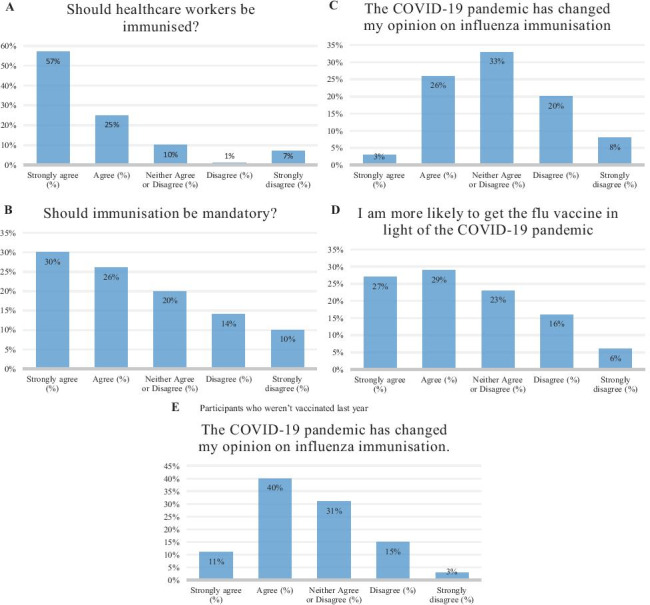
Results of Likert-based questions

A total of 719 participants answered this question, the majority of whom strongly agreed with this statement (57%, *n* = 408). A further 25% (*n* = 182) agreed, 10% (*n* = 72) could neither agree nor disagree, 1% (*n* = 10) disagreed and 7% (*n* = 47) strongly disagreed (Fig. 2A). In 2018, 383 participants answered this question. The majority strongly agreed (56%, *n* = 213) with a further 28% (*n* = 106) agreeing, 8% (*n* = 30) neither agreeing nor disagreeing, 0.3% (*n* = 1) disagreeing and 9% (*n* = 33) strongly disagreeing.

### I believe influenza immunisation for healthcare workers should be mandatory

A total of 718 participants answered this question, with 26% (*n* = 189) agreeing with this statement and a further 30% (*n* = 216) strongly agreeing (Fig. 2B). Twenty percent (*n* = 141) could neither agree nor disagree with this statement. Fourteen percent (*n* = 99) disagreed with another 10% (*n* = 73) strongly disagreeing. In 2018, 392 participants answered this question, with 26% (*n* = 103) agreeing with this statement and a further 29% (*n* = 114) strongly agreeing. Twenty-two percent (*n* = 87) could neither agree nor disagree with this statement. Fourteen percent (*n* = 53) disagreed with another 9% (*n* = 35) strongly disagreeing.

### The COVID-19 pandemic has changed my opinion on influenza immunisation

A total of 722 participants answered this question (Fig. 2C). The majority of participants could neither agree nor disagree that the COVID-19 pandemic had changed their opinion of influenza immunisation (33%, *n* = 238). Twenty-six percent (*n* = 190) of participants agreed with this statement, with a further 13% (*n* = 95) strongly agreeing. Twenty percent (*n* = 141) disagreed and 8% (*n* = 58) strongly disagreed.

When focusing on those who were not vaccinated last year (*n* = 159), 158 participants answered this question (Fig. 2E). The majority of whom agreed that the COVID-19 pandemic had changed their opinion on influenza immunisation (40%, *n* = 63), with another 11% (*n* = 18) strongly agreeing. Thirty-one percent (*n* = 49) could neither agree nor disagree with this statement. Fifteen percent (*n* = 24) disagreed with a further 3% (*n* = 4) strongly disagreeing.

### I am more likely to get the influenza immunisation in light of the COVID-19 pandemic

A total of 710 participants answered this question (Fig. 2D). Twenty-eight percent (*n* = 202) of participants agreed that they were more likely to get the vaccine in light of COVID-19, with 27% (*n* = 193) strongly agreeing. Twenty-three percent (*n* = 162) could neither agree nor disagree with this statement. Sixteen percent (*n* = 112) disagreed with another 6% (*n* = 41) strongly disagreeing.

### Suggestions for increasing staff uptake

**Fig. 3:**
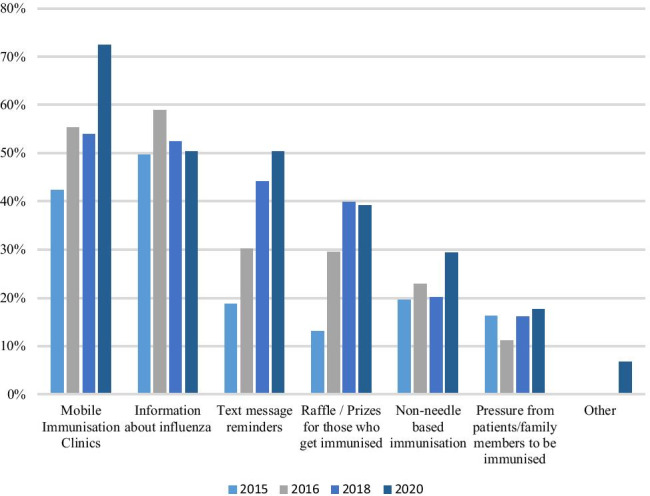
Suggestions for increasing uptake amongst healthcare workers

Of the suggestions provided to participants to help increase staff uptake of the vaccine, 72% agreed that more mobile immunisation clinics would benefit. Over half of participants agreed that more information on the vaccine (50%) and text reminders about the immunisation clinics (50%) would increase uptake. Thirty-nine percent chose raffle prizes for those who get vaccinated, with 29% choosing non-needle-based vaccination. These results are compared to previous years’ results in Fig. 3. This year was the first year that mobile immunisation clinics surpassed information on the vaccine as the most popular suggestion.

### Comments

Participants were also given the opportunity to provide comments at the end of the survey. The main suggestion was the need for more information and education (*n* = 15). Participants asked for education on the development of the vaccine, its safety and efficacy, side effects, the risks and benefits of the vaccine. A need to dispel the myths surrounding the vaccine was expressed by three participants. Methods of increasing education included a mandatory lecture for staff or showing a video of how the flu is spread from healthcare workers onward.

Convenience was mentioned by three participants, through the use of peer vaccinators and vaccination in places of convenience in the hospital. Two participants mentioned peer pressure as a promoter of vaccination while three mentioned pressure from management. One suggestion was that consultants encouraged and even went with their teams to be vaccinated. Two comments mentioned rewards in the form of vouchers or food. Three comments involved the need for more scientific evidence to support the flu vaccine, with one requesting data from other hospital sites to compare this institution to, with regards to staff uptake.

In terms of barriers, two participants mentioned limited vaccination times as a barrier to them being vaccinated in the past. Two participants voiced concern around side effects and getting the flu from the vaccine. One participant reported anaphylaxis as a barrier to being vaccinated. While some participants described the need for the vaccine to be mandatory (*n* = 3), others expressed that we all deserve the right to choose to get vaccinated (*n* = 3).

### The flu vaccine campaign strategies

Following the results of this year’s audit, a number of strategies were added to the flu vaccine campaign in this university hospital. Education was addressed by supplying staff access to the HSEland online module on the flu vaccine [10]. Feasibility was addressed by the implementation of a streamline process for staff to be vaccinated. Staff could sign up for an appointment in advance via an online booking system. Previously, staff were advised to stay in the Occupational Health Department for 15 min post-vaccination to monitor for allergic reactions. This rule was amended, with staff needing to stay on the hospital premises for at least 15 min after being vaccinated. This allowed for staff to return to their work much sooner. Peer vaccination was emphasised this year and a number of different types of staff volunteered (i.e., nurses and doctors). The uptake rate this year was much higher than previous years. In the first day of the campaign, there were over 800 vaccinations. This increased to 1900 in the first week, exceeding the 1100 vaccinations in the first month of 2019′s flu campaign. Over 54% of staff were vaccinated during week 1 of the campaign. In 2018–2019, 41% of hospital staff were vaccinated in this hospital in total.

## Discussion

The barriers to vaccination in this study remain to be feasibility, perceived side effects of the vaccine and not believing that the vaccine works. Feasibility presented the most common barrier to staff this year, compared with side effects in previous years. The importance of feasibility was much greater this year than previously demonstrating a shift in attitude of staff towards vaccination. This was addressed via a streamline online process for staff to be vaccinated. Literature attempting to identify the reasons for hesitancy towards vaccination has pointed to low-risk perception, negative attitudes toward vaccination in general, lack of adequate influenza-specific knowledge and lack of access to vaccination facilities as some of the negative predictors of vaccine uptake [11]. With the implementation of numerous strategies, staff uptake rates have increased significantly in our institute, exceeding the total uptake rate last year, in the first week of this year’s campaign.

Multicomponent strategies have been found to be more effective at increasing vaccination rates rather than single component strategies [12, 13]. Education alone has not been shown to increase vaccination rates significantly [14]. A study in Japan managed to increase their uptake rates to 97% through a multifaceted intervention including the use of a declination form, free vaccination, hospital-wide announcements about the vaccination campaign, prospective audit and real-time telephone interview for healthcare workers who were not vaccinated [15].

Over 50% of participants agreed that influenza immunisation should be mandatory. A hospital in Seattle implemented a mandatory influenza immunisation programme [16]. A total of 97.6% of staff were vaccinated and these figures were maintained over the subsequent 4 years of the study. A similar intervention was taken in Canada, which saw a 74% uptake rate [17]. In both studies, staff who refused to be vaccinated were mandated to wear a mask on the wards. Influenza immunisation has not been made mandatory in this hospital as yet; however, due to the COVID-19 pandemic, staff are required to wear a face covering in a healthcare setting in Ireland by law [18].

As part of the flu vaccine campaign, peer vaccinators administered the vaccines to staff according to the Health Service Executive’s (HSE) Seasonal Influenza Peer Vaccination Programme [19]. Peer vaccinators are registered nurses, registered midwives or registered medical practitioners with basic life support training and training in anaphylaxis management. This increased the number of people able to vaccinate staff, thus improving the feasibility of being vaccinated with reduced waiting times.

This year, it is crucial that healthcare workers are vaccinated against influenza, to reduce the burden on the hospital amid the COVID-19 pandemic, which has also been called a twindemic [20]. Healthcare workers should be vaccinated to protect their vulnerable patients [21]. Despite the implications of a twindemic, rates of influenza in 2020 have remained historically low in both Northern and Southern Hemispheres [22]. The global decline in influenza rates appears to be associated with community mitigation measures against COVID-19 such as social distancing, mask wearing and staying at home when sick. These measures may be useful adjuncts to influenza immunisation in future influenza epidemics to reduce transmission.

The results of the Likert-based questions in this survey indicated that staff were more likely to be vaccinated in light of the COVID-19 pandemic. The COVID-19 pandemic has provided a unique opportunity for maximizing uptake rates of influenza immunisation. This attitude of HCWs is not only important in terms of tackling COVID-19 when a vaccine is approved, but also a stepping stone for immunisation campaigns in future years. A national survey took place in the USA on attitudes towards a potential SARS-CoV-2 vaccine on 1000 US adults. A total of 57.6% of participants intended to be vaccinated, 31.6% were not sure and 10.8% did not intend to be vaccinated [23]. Having not received an influenza vaccine last year was independently associated with vaccine hesitancy. The most common reasons for vaccine hesitancy amongst those who were “unsure” were having specific concerns about the vaccine and needing additional information, compared with holding anti-vaccine attitudes/beliefs/emotions and lack of trust amongst those who did not intend to get the vaccine. Since 42.4% of participants were hesitant to accept vaccination against COVID-19, it is clear that education and information on vaccine safety are needed to achieve uptake rates once a vaccine is developed.

### Strengths and limitations

While the COVID-19 pandemic does appear to have some influence on HCW influenza immunisation, one strength of this study is that the results were compared to the pre-COVID era.

One limitation is that this survey took place in a single institution, so findings may lack generalisability. Another limitation is that participation in this survey was voluntary and thus may introduce selection bias, as survey participants may be more interested in flu vaccination.

## Conclusion

In light of the increasing number of survey participants, more staff were interested in flu vaccination this year than ever before. The COVID-19 pandemic has had some influence on staff’s likelihood to be vaccinated. Feasibility of immunisation and education posed the largest barriers to HCW vaccination. With the implementation of a streamline vaccination programme including peer vaccinators, uptake rates have increased significantly since the beginning of 2020′s flu campaign.

## Data Availability

The data collected on sphinx is not available online.

## Appendix 1. Survey questions

What is your gender?

Male

Female

Other

Age

Under 20

20–29

30–39

40–49

50–59

60–69

What is your occupation? (Tick all that apply)

Doctor

Nurse

Allied health professional

Management/administration

General support services

Other patient care (i.e. HCA)

Did you get the influenza immunisation last year?

Yes, I got it.

No, I did not get it.

If yes, why did you get it? (Tick all that apply).

It’s what’s recommended.

Protect my patients.

Protect family members.

Protect myself from becoming unwell.

Protect colleagues.

Other members of staff are doing it.

Will you get the influenza immunisation this year?

Yes, I will get it.

No, I will not get it.

If not, why not? (Tick all that apply).

Unable to find time.

I believe it gives me the flu.

I don’t think it works.

I can manage the flu myself.

Immunisation sites are not conveniently located.

I don’t like the needles/arm/pain.

I am worried about side effects (apart from pain at the injection site).

I believe healthcare workers should be immunised against influenza.

Strongly disagree.

Disagree.

Neither agree nor disagree.

Agree.

Strongly agree.

I believe influenza immunisation for healthcare workers should be mandatory.

Strongly disagree.

Disagree.

Neither agree nor disagree.

Agree.

Strongly agree.

What suggestions do you think would help increase staff uptake of influenza immunisation? (Tick all that apply).

Information about influenza.

Raffles/Prizes for those who get immunised.

Text message reminders of immunisation clinics.

Mobile immunisation clinics coming to wards/other areas.

Non-needle based immunisation.

Pressure from patients/family members to be immunised.

Other (please specify).

The COVID-19 pandemic has changed my opinion on influenza immunisation.

Strongly disagree.

Disagree.

Neither agree or disagree.

Agree.

Strongly agree.

I am more likely to get the influenza immunisation in light of the COVID-19 pandemic.

Strongly disagree.

Disagree.

Neither agree nor disagree.

Agree.

Strongly agree.

## Code availability

The software Clinical Audit Sphinx was used for data collection and analysis.

## References

[1] To KW, Lai A, Lee KC, Koh D, Lee SS (2016). Increasing the coverage of influenza vaccination in 272 healthcare workers: review of challenges and solutions. J Hosp Infect.

[2] G Dini A Toletone L Sticchi A Orsi NL Bragazzi P Durando 2017 Influenza vaccination in 274 healthcare workers: A comprehensive critical appraisal of the literature Hum Vaccin Immunother 275 10.1080/21645515.2017.1348442276 10.1080/21645515.2017.1348442 PMC5861785 28787234

[3] World Health Organization (2015) Influenza (2015). Available 277 from http://www.who.int/topics/influenza/en. [Accessed 31st Octboer 2020] 278

[4] Taylor G, Mitchell R, McGeer A (2015). Healthcare-associated influenza in Canadian hospitals 279 from 2006 to 2012. Infect Control Hosp Epidemiol DOI.

[5] Kawana A, Teruya K, Kirikae T, et al. “Syndromic surveillance within a hospital” for the early 281 detection of a nosocomial outbreak of acute respiratory infection. Jpn J Infect Dis 2006;59:377e379. 282 17186956

[6] Ridgway JP, Bartlett AH, Garcia-Houchins S (2015). Influenza among afebrile and vaccinated 283 healthcare workers. Clin Infect Dis.

[7] Barberis I, Myles P, Ault SK, Bragazzi NL, Martini M (2016) History and evolution of influenza 285 control through vaccination: from the first monovalent vaccine to universal vaccines. J Prev Med Hyg. 286 2016;57(3):E115-E20. PubMed PMID: 27980374. 287 PMC5139605 27980374

[8] Health Protection Surveillance Centre (2019) Uptake of the Seasonal Influenza Vaccine in Acute 288 Hospitals and Long Term/Residential Care Facilities in Ireland in 2018‐2019. https://www.hpsc.ie/a-289 z/respiratory/influenza/seasonalinfluenza/influenzaandhealthcareworkers/hcwinfluenzavaccineuptakere290 ports/Seasonal%20Influenza%20Vaccine%20Uptake-2018–2019.pdf [Accessed November 1st 2020] 291

[9] Health Services Executive (2020) Why influenza vaccination is important for healthcare workers 292 (HCWs). September 2020. https://www.hse.ie/eng/health/immunisation/pubinfo/flu-293 vaccination/whyflunb4hcws.pdf [Accessed 31st October 2020] 294

[10] Health Service Executive (2020) The flu vaccine – protect yourself, protect others [Internet]. 295 Hseland.ie. Available from: 296 https://www.hseland.ie/ekp/servlet/ekp?PX=N&TEACHREVIEW=N&PTX=&CID=EKP000000150&297 TX=FORMAT1&LANGUAGE_TAG=0&DECORATEPAGE=N [accessed 11 November 2020]. 298

[11] Schmid P, Rauber D, Betsch C, Lidolt G, Denker ML (2005) Barriers of Influenza Vaccination 299 Intention and Behavior - A Systematic Review of Influenza Vaccine Hesitancy, 2005 - 2016. PLoS 300 One. doi: 10.1371/journal.pone.0170550. 301 10.1371/journal.pone.0170550 PMC5268454 28125629

[12] Dubé E, Gagnon D, MacDonald NE (2015). Strategies intended to address vaccine hesitancy: review 302 of published reviews. Vaccine.

[13] Ndiaye SM, Hopkins DP, Shefer AM, Hinman AR, Briss PA, Rodewald L, Willis B (2005) 304 Interventions to improve influenza, pneumococcal polysaccharide, and hepatitis B vaccination 305 coverage among high-risk adults: a systematic review. American journal of preventive medicine. 2005 306 Jun 1;28(5):248–79. 307 10.1016/j.amepre.2005.02.016 15894160

[14] Llupià A, Mena G, Olivé V, Quesada S, Aldea M, Sequera VG, et al (2013) Evaluating influenza 308 vaccination campaigns beyond coverage: a before-after study among health care workers. Am J Infect 309 Control. doi: 10.1016/j.ajic.2013.04.006. 310 10.1016/j.ajic.2013.04.006 23896285

[15] Honda H, Sato Y, Yamazaki A, Padival S, Kumagai A, Babcock H (2013) A successful strategy for 311 increasing the influenza vaccination rate of healthcare workers without a mandatory policy outside of 312 the United States: a multifaceted intervention in a Japanese tertiary care center. Infect Control Hosp 313 Epidemiol. doi: 10.1086/673452 314 10.1086/673452 24113604

[16] Rakita RM, Hagar BA, Crome P, Lammert JK (2010). Mandatory influenza vaccination of healthcare 315 workers: a 5-year study. Infect Control Hosp Epidemiol doi.

[17] Ksienski DS (2014). Mandatory seasonal influenza vaccination or masking of British Columbia health 317 care workers: year 1. Can J Public Health.

[18] Health Service Executive (2020) Face coverings, medical masks and disposable gloves [Internet]. 319. Available from: https://www2.hse.ie/conditions/coronavirus/face-masks-disposable-320 gloves.html [cited 10 November 2020]. 321

[19] National Immunisation Office, National Clinical Lead in Occupational Health (2019) Seasonal 322 Influenza Peer Vaccination Programme Guidelines for Staff. Available at: 323 https://www.hse.ie/eng/health/immunisation/hcpinfo/fluinfo/peerguidelines.pdf [Accessed 12th 324 November 2020] 325

[20] Hoffman J. (2020) Fearing a ‘Twindemic,’ Health Experts Push Urgently for Flu Shots [Internet]. 326 Nytimes.com. 2020 Available from: https://www.nytimes.com/2020/08/16/health/coronavirus-flu-327 vaccine-twindemic.html [Accessed 13 November 2020]. 328

[21] Music T (2012). Protecting patients, protecting healthcare workers: a review of the role of influenza 329 vaccination. Int Nurs Rev.

[22] SJ Olsen E Azziz-Baumgartner AP Budd L Brammer S Sullivan RF Pineda C Cohen AM Fry 331, 2020 Decreased influenza activity during the covid-19 pandemic—United States, Australia, Chile, 332 and South Africa, 2020 Morb Mortal Wkly Rep 10.1111/ajt.16381333 10.15585/mmwr.mm6937a6 PMC7498167 32941415

[23] KA Fisher SJ Bloomstone J Walder S Crawford H Fouayzi KM Mazor 2020 Attitudes toward a 334 potential SARS-CoV-2 vaccine: a survey of US adults Ann Intern Med 335 10.7326/M20-3569 10.7326/M20-3569 PMC7505019 32886525

